# Improved real-time blood flow velocity quantification via application of the Karhunen-Loeve transform for increased signal-to-noise ratio

**DOI:** 10.1186/1532-429X-13-S1-P38

**Published:** 2011-02-02

**Authors:** Samuel Ting, Yu Ding, Orlando P Simonetti

**Affiliations:** 1 The Ohio State University, Columbus, OH, USA

## Objective

Evaluate the application of temporal filtering via the Karhunen-Loeve Transform for increasing signal-to-noise ratio in real-time blood flow quantification.

## Background

Real-time blood flow velocity quantification requires fast acquisition time and both high spatial and temporal resolution in order to capture clinically relevant features. Multi-coil parallel imaging techniques trade fast real-time cine acquisition with high temporal resolution for lower signal quality. Methods for increasing signal-to-noise ratio (SNR) will allow higher acceleration rates in parallel imaging, leading to faster acquisition times in the context of exercise stress while maintaining signal quality.

Increased SNR via the Karhunen-Loeve Transform [1] (KLT) is achieved by decomposing a temporal cine sequence into a series of eigenmodes where the signal of interest is localized in a subset of eigenmodes and eliminating noise-only eigenmodes.

## Methods

KLT was applied retrospectively to ten through-plane real-time velocity data sets of the aorta at rest acquired at 1.5T (Avanto, Siemens, Malvern, PA). Optimal identification of noise-only eigenmodes was determined using the Marcenko-Pastur Law [2], and SNR gain was calculated as the square root of the ratio of noise-only eigenmodes to total number of eigenmodes.

## Results

**Figure 1:**
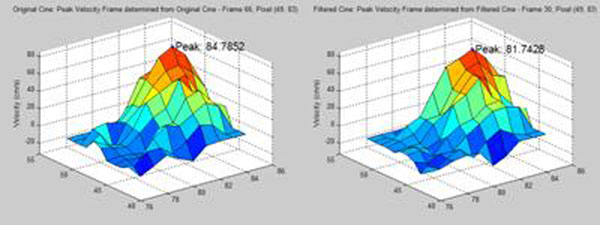
Velocity profiles showing peak velocity measurement for original real-time cine (left) and KLT filtered cine (right).

Optimal selection of SNR gain via the Marcenko-Pastur Law results in SNR increase of 61.5 ± 3.7% (mean ± standard deviation) with no statistically significant difference in peak velocity measurement (Difference: 1.45 ± 2.98%, p-value: 0.2794). Figure 1 illustrates the minimal difference in peak velocity measurement due to KLT filtering.

## Conclusions

Temporal filtering of real-time velocity images via KLT results in significant gains in SNR with minimal velocity profile smoothing and minimal effect on peak velocity measurement and may be useful in improved blood flow velocity quantification.

## References

[B1] Phys. Med. Biol 2009 54 3909 10.1088/0031-9155/54/12/020 19491455

[B2] Mag 2010 63 782 789 10.1002/mrm.22258

